# “I wish I had known what I was getting into”: a qualitative study exploring the experiences of Canadians who study medicine abroad

**DOI:** 10.1186/s12909-023-04367-1

**Published:** 2023-05-24

**Authors:** Maria Mathews, Dana Ryan, Ivy Bourgeault

**Affiliations:** 1 Department of Family Medicine, Schulich School of Medicine & Dentistry, Western University, 1151 Richmond St., London, ON N6A 5C1 Canada; 2 School of Sociological and Anthropological Studies, Faculty of Social Sciences, University of Ottawa, Ottawa, Canada

**Keywords:** Medical school, Canadian’s studying abroad, Residency selection, Post-graduate training, International medical graduates, Qualitative research

## Abstract

**Background:**

An increasing number of Canadians are choosing to study medicine abroad (CSA); however, many CSA are not fully informed of the challenges that exist in returning to Canada to practice and relatively little information is known on the topic. This study explores CSA experiences in choosing to study abroad and their attempts to navigate a return to Canada to practice medicine.

**Methods:**

We conducted semi-structured qualitative interviews with CSA who were attending medical school abroad, waiting to obtain or in a post-graduate residency program, or practicing in Canada. We asked participants about their decision to study medicine abroad and choice of school, medical school experiences, activities they engaged in to increase their likelihood of returning to Canada, perceived barriers and facilitators, and alternative plans if they were unable to return to Canada to practice. Interviews were transcribed and analyzed using a thematic analysis approach.

**Results:**

Fourteen CSA participated in an interview. Expedited timelines (i.e., direct entry from high school) and a lack of competitiveness for medical school in Canada were the main justifications for CSAs’ decision to study abroad and a number of key factors (e.g., location, reputation) influenced their choice of school. Participants reported not fully anticipating the challenges associated with obtaining residency in Canada. CSA relied upon a variety of informal and formal supports and employed numerous methods to increase their likelihood of returning to Canada.

**Conclusions:**

Studying medicine abroad remains a popular choice for Canadians; however, many trainees are unaware of the challenges associated with returning to Canada to practice. More information on this process as well as the quality of these medical schools is needed for Canadians considering this option.

**Supplementary Information:**

The online version contains supplementary material available at 10.1186/s12909-023-04367-1.

## Background

Canadians who choose to study medicine outside Canada (known as Canadians who study medicine abroad – CSA) take an enormous risk because they are often ill-informed of the steps and requirements for returning to Canada to practice medicine. Persuaded by the appealing messaging from entrepreneurial medical schools [1–3], CSA are often unaware that offshore medical schools abroad offer few opportunities for CSA to complete post-graduate training in the host country [4] or that there is steep competition for the limited number of post-graduate positions available to international medical graduates [4–7] (IMG; defined as any physician who, regardless of citizenship or permanent residency status in Canada, graduated from medical school outside Canada, or for post-graduate training purposes, the United States [US]) [8, 9]. Without post-graduate training, CSA are unable to practice in either Canada or in the host country. For over 30 years, substantial numbers of Canadians have turned to studying medicine outside Canada [1–3, 6, 7, 10]. While CSA comprise a growing proportion of trainees in post-graduate residency programs in Canada, concerns about the quality of medical education in these international programs persist [1, 11–14].

Media reports create a sympathetic portrait of CSA as well-qualified applicants who were turned away from medical schools in Canada, and after studying medicine abroad, are unable to return to Canada to practice because of limited post-graduate residency positions or other bureaucratic hurdles [1–3, 15–17]. CSA are often touted as a solution to Canada’s physician shortages – a message that document analyses have shown is featured predominantly in the websites of offshore medical schools [14, 16, 17]. A survey of CSA found that, compared to Canadian medical graduates, a larger proportion of CSA are male, older, have more post-secondary education, have a physician as a parent, and applied fewer times for admission to medical schools in Canada [18]. Most CSA have few opportunities to complete post-graduate training where they went to medical school and therefore decide to return to Canada to complete post-graduate training and practice [18]. A judicial review of selection processes for post-graduate medical residency programs in Ontario (Canada’s most populated province) concluded that some screening criteria (such as recent graduation from medical school and clinical experience in Canadian settings) benefit CSA over immigrant IMG [7]. Analyses of administrative data on post-graduate medical residents found that CSA were almost five times as likely as immigrant IMG to obtain a post-graduate position [16]. There was, however, no difference in the examination success rates of CSA and immigrant IMG post-graduate trainees admitted to residency positions in Canada, or their subsequent work locations [19], countering suggestions in media reports that CSA are easier to train than immigrant IMG and offer a potential solution for physician shortages.

In light of these conflicting narratives, we conducted qualitative interviews with CSA to explore their first-hand experiences in choosing to study abroad and their attempts to navigate a return to Canada to practice medicine. Despite the growing popularity of studying medicine abroad, there is relatively little information about the experiences of CSA to inform prospective students, their families, or medical educators in Canada who may place too much emphasis on publicity materials and media reports [14].

## Methods

Using a pragmatic approach, we conducted a descriptive qualitative study using semi-structured interviews with CSA who were in medical school abroad, completed medical school but waiting to obtain a residency position, in a post-graduate residency program, or practicing in Canada. To be included in the study, CSA had to have been Canadian citizens or permanent residents before enrolling in medical schools outside Canada or the US and wanting to return to Canada to practice. We excluded US medical graduates because the Canadian Residency Matching Service (CaRMS) currently treats US medical graduates as equivalents to Canadian medical graduates in matching for post-graduate residency positions [8, 9].

To recruit participants, we asked administrators at overseas medical programs to email students a study invitation. We asked the Society of Canadians who Study Abroad to email study invitations to its members and post the study invitation on its Facebook page. The post-graduate programs at Memorial University and the University of Ottawa (where the authors were based) emailed study invitations to its post-graduate medical trainees. We posted recruitment banners on the Canadian Health Workforce Network and Health Worker Migration websites, and the social media accounts affiliated with these groups. We also asked study participants to inform colleagues and friends about the study (i.e., snowball sampling) and continued recruitment until we reached saturation along main themes [20, 21].

Study invitations asked interested CSA to contact a research assistant who provided additional study information and obtained consent, and the three authors (based in Newfoundland and Labrador and Ontario, Canada) conducted interviews by phone or in-person in English. We asked participants about (1) their decision to study medicine abroad and the factors that influenced their choice of schools; (2) their stage in the licensing process; (3) their medical school experiences; (4) the activities they undertook to improve their ability to return to Canada to practice; (5) their perceptions of the barriers to and facilitators for returning to Canada to practice; and (6) their plans if they were unable to return to Canada (Appendix A). Questions were tailored to the CSA’s career stage. We also gathered relevant demographic data.

Fifteen CSA contacted a research assistant to express interest in participating in the study and 14 (93.3%) completed an interview. One person did not respond to invitations to arrange a time for an interview. Twelve interviews were done by phone and two were done in-person. The interviews, conducted in English, were 26 to 64 minutes long (mean 40 minutes).

Interviews were recorded and transcribed verbatim. Using a thematic analysis approach, two members of the research team (MM and DR) independently read six transcripts in order to identify key words and codes, which we organized into a preliminary coding scheme, then compared to refine and develop a unified and robust coding scheme [20, 21]. The final coding template was used to code all of the transcripts using NVIVO (software designed to assist in the organization and management of qualitative data). We compared across career stages (pre-residency, residency, practice) and gender during the analysis. The major themes are presented in the results. Frequencies were used to summarize participant demographic data.

We took a number of steps to ensure the rigour of our analyses [20–23]. During each interview, we summarized and reflected responses back to ensure we understood the meaning of participant responses. We kept detailed records of the interview guides, digital recordings, transcripts, field notes, drafts of the coding template, and coding disagreements and their resolutions. We looked for negative cases and provide thick description and illustrative quotes.

### Positionality

The authors are health workforce researchers with more than twenty years experience examining the migration, regulation, and integration of physicians in Canada’s health workforce. Using a pragmatic approach, we designed the study to understand how CSA navigate the system, with an intention to understand CSA experiences within the policy context for training and licensing IMG in Canada. To this end, we recruited participants at various career stages and asked about their interactions with the medical education and regulatory system in Canada.

## Results

The characteristics of the participants are summarized in Table 1.

Eight (57.1%) of the CSA were not yet in a residency program, 4 (28.6%) were in a residency program, and 2 (14.3%), were practicing physicians. There were 5 men and 9 women participants. All participants had grown up in Canada and were Canadian citizens. Nine (64.3%) attended medical school in Ireland, 3 (21.4%) in the United Kingdom (UK), 1 (7.1%) in Grenada, and 1 (7.1%) in Australia.

**Table 1:** Characteristics of Interview Participants

Characteristics	n %
Career Stage	
In medical school abroad In residency program in Canada Practicing in Canada	8 (57.1) 4 (28.6) 2 (14.3)
Gender*	
Male Female	5 (35.7) 9 (64.3)
Birthplace	
Canada Outside Canada	12 (85.7) 2 (14.3)
Country where they grew up	
Canada	14 (100)
Citizenship	
Canada	14 (100)
Location undergraduate degree**	
Canada None (direct entry medical program)	9 (64.3) 5 (35.7)
Country of Medical School	
Australia Grenada Ireland United Kingdom	1 (7.1) 1 (7.1) 9 (64.3) 3 (21.4)

*self-reported; **prior to medical school

Participants’ reasons for choosing to study medicine abroad largely fell into two general themes: they wanted to enter medical school directly from high school or they were unable to get admitted into a medical school in Canada. Most study participants knew early in their academic career that they wanted to become physicians and wanted the certainty of proceeding directly into medical school: “*I chose to go to medical school in the UK because I could go straight into medical school from high school. I knew what I wanted to do and I wasn’t willing to … wait to get into medical school*” [25; Pre-residency; UK]. These participants were attracted to the 6-year direct entry program that was offered by schools in the UK and Ireland that allowed them to avoid completing a 4-year undergraduate degree, which many of them viewed as an unnecessary and time-consuming requirement to studying medicine in Canada: “*I didn’t want to wait in Canada to do an undergraduate degree and then medical school. So Ireland offered a 6 year program from high school*” [9; Post-residency; Ireland]. CSA also opted to study medicine abroad because they were not admitted to medical school in Canada. Some had applied many times and been interviewed (“*I applied to the University of British Columbia five years in a row and interviewed five years in a row. Never got in.”* [22; Residency; Ireland]) while others had not applied to medical school in Canada because they knew that they were not competitive (“*I wouldn’t meet [the grade point average] cut-off, and so I didn’t even apply*” [14; Post-residency; Grenada]). One participant noted that he knew he would not be a competitive applicant because he decided to study medicine late in his academic career and felt that he did not fit the typical mould of a medical student: “*I thought about medical school just towards the end of my first undergraduate degree… I suppose, if you’re trying to get into medical school, you really sort of decide this when you start your degree and you tailor your whole degree towards it*” [19; Residency; Ireland].

CSA in our study learned about international medical schools from a variety of sources. Two study participants cited an article in MacLean’s [16], a Canadian-based current affairs monthly magazine: “*I read a MacLean’s article… on how many Canadians study medicine abroad … And so, my dad and I kind of sat down and looked up some of the different schools and just kind of on a whim decided to apply to Australia*” [11; Pre-residency; Australia]. Other participants heard about medical schools that accepted students from Canada through word of mouth from family members and friends who knew someone who had studied at these institutions: “*I had heard about St. George’s through a family friend who went there”* [14; Practice; Grenada]. For participants who went to medical school directly from high school, counsellors were an influential source of information (“*It was my high school guidance counselor who said, ‘oh, maybe you should think about applying overseas…’*” [9; Practice; Ireland]). Participants usually consulted the websites of individual schools to learn more about the programs and the application and admission process. Many noted that, in hindsight, they did not have a full appreciation of the difficulty in returning to Canada to work: *“I wish I had known what I was getting into before I came out here… [it] would have been nice to have had some information provided about it”* [6; Pre-residency; Ireland]. They reported that there was a need for more information on the barriers to working in Canada and noted the risk of pursuing medical education abroad without fully understanding these barriers: “*People are going overseas not realizing the barriers to coming back*” [9; Practice; Ireland].

CSA in our study chose schools based on a number of factors, including ease of travel, training in English, and culture: “*I know that the culture is pretty similar to the culture in Canada and like, the language is similar. And even though it’s in Europe, it’s still … it’s not too far. It’s a five-hour flight from the East Coast of Canada*” [22; Residency; Ireland]. They also considered costs (from tuition, travel, living expenses, and examination fees): “*There was a lot of expenses with every exam, flying to different places, so the financial burden was very extreme*” [6; Pre-residency; Ireland]. Participants also valued the school’s general reputation, particularly in regard to preparing students for residency programs in Canada and the United States: “*I selected Ireland first before I would have considered Australia or the Caribbean because… they have the longest history of sending doctors back to Canada and have the sort of highest success rate of matching residents back to Canadian programs*” [19; Residency; Ireland].

Participants commented on the relative ease with which they were able to apply and gain acceptance into these schools. Applicants may not be required to complete standardized examinations such as the Medical College Admission Test (MCAT) (“*There was no requirement for MCATs*” [1; Residency; Ireland]) or interviews (“*I did not have an interview for my medical school*…” [22; Residency; Ireland]) and admission requirements are perceived to be lower than those of medical schools in Canada (“*I guess, having not gotten into the ones [in Canada] and having gotten in [in Ireland], I would assume their admission requirements are a bit easier*” [3; Residency; Ireland]). Participants also commented on the relative ease and efficiency of application processes which may be facilitated by intermediary companies (“*It’s like a company that takes in all your applications, forwards them to the school, takes your deposit, schedules your interviews. So that was certainly helpful for the application process. They also gave advice as to travel, accommodation…*” [27 Pre-residency; Ireland]) and the shorter time to admission than Canadian medical schools, which take roughly an academic year *(*“*…from application to acceptance was about one month*” [11; Pre-residency; Australia]).

We also asked participants to comment on how their experiences in medical school abroad compared to Canadian medical schools. Participants described the first two years in a four-year program (or first four years in a six-year, direct-entry program) as similar to most pre-clerkship years in medical schools in Canada and used a body-systems approach and/or problem-based learning. During clerkship, participants’ experiences varied by location. Participants who attended medical schools in Ireland, the UK, or Australia noted that they had much less hands-on responsibility for managing patients than in Canada, and that their role was primarily to observe:

*I’d say the greatest shortcoming of Ireland would be that during your clinical years you’re not given much responsibility, as opposed to the medical clerks or medical team. The medical students [in Canada], who are given their own patients and have a very defined role on the team. There you’re more so an observer, for the most part.* [3; Residency; Ireland]

A participant who studied in the Caribbean noted that clinical training was done in the United States: “*being in the United Sates for my latter two years … it was great to learn the American way of practice … because I wouldn’t really say that we had much clinical exposure in Grenada, like, we weren’t in the hospitals treating the Caribbean patients*” [14; Post-residency; Grenada].

Participants also noted that they had to juggle completing paperwork (travel documents, applications and approvals for electives, credentials, and loans and finances) during medical school: “*There’s a lot of paperwork involved … letters from the Dean approving your elective, letters from the [police service] saying that you have a clean criminal record. … Every time you have a new holiday and a new set of electives that you have to do, you have to get a new set of these documents*” [19; Residency; Ireland]. Participants also had to manage logistics to ensure that they were able to write the necessary qualification examinations, and apply for electives and vacations: “*you have to schedule yourselves for electives during med school back at Canadian medical schools and that is a bit of a challenge in scheduling because you’re filling up all of your holiday time to get as many of those electives done as you can*” [19; Residency; Ireland].

Participants noted the need to schedule carefully to meet deadlines, especially for events that were held once every year (e.g., National Assessment Collaboration Objective Structured Clinical Examination [NAC OSCE], CaRMS deadlines) or those that delayed the ability to apply for residency training if missed:

*I remember in my final year I was flying back and forth between North America and Ireland continuously doing my exams in between my rotations. And just to throw the Canadian exams into the loop and then for the new group of people having to do the NAC OSCE which, if you’re on that one rotation where you just can’t get off, time off, you lose an entire year.* [1; Residency; Ireland]

Because CSA may apply for post-graduate training in more than one country, participants needed to understand and stay on top of multiple, concurrent requirements, forms, and deadlines while also completing their final year of medical school.

Participants quickly realized that obtaining a residency position is very competitive and described a number of methods they used to increase their likelihood of being accepted into a training program. Most participants completed electives in Canada as part of their training or during their breaks from medical school. They noted that familiarizing themselves with the Canadian health care system and potential training programs and obtaining reference letters from faculty members at Canadian medical schools would be viewed favourably in the selection process:

*So these electives are perfect for that because you get your letters of recommendation from a clinical standpoint, from some of the attendants that you would do these electives at and program directors … when you’re doing a personal statement and stuff, you want to be able to say that you’ve been there so you can talk about certain things that you really enjoyed and things that … attract you to the program.* [26; Pre-residency; Ireland]

Participants were aware of their limited choice of specialty programs and sometimes opted to choose programs that increased the likelihood of a match as opposed to selecting programs based on their area of interest: “*you’re limited in what specialties you have options in, because there’s specific IMG spots and they may not necessarily pertain to every specialty*” [9; Post-residency; Ireland]. Participants also applied to post-graduate programs in multiple countries where they may be eligible as some CSA recognized that they may not be able to return to Canada: “*I also applied to the States as well, that’s why I did the USMLE [United Stated Medical Licensing Exam] exams. But ah, so in terms of hierarchy it would have been Canada, America, Ireland, and then cry in a corner*” [1; Residency; Ireland].

During their studies, participants benefitted from various supports, including informal support from upper classmates: “*I was alongside a big group of Canadians studying abroad and it was so nice to have. Because they all knew when to renew the visa, when to apply for the Board exam, and just making sure that you’re on top of everything from the Canadian standpoint”* [14; Post-residency; Grenada]. They also relied on in-person and online peer support, and websites *(*“*…there certainly are chat forums online through a variety of websites, …And those forums tend to be quite active*” [3; Residency; Ireland]); student organizations at their school or country of study (“*we had a student organization …who helped in terms of organizing events to teach us how to navigate [the process to return to Canada]*” [1; Residency; Ireland]); as well as formal resources in Canada such as advocacy groups and residency preparation: “*There is an association in Toronto, who do offer free interview help for IMGs … They were able to do a few mock interviews for me through Skype and they gave me feedback based on those interviews*” [3; Residency; Ireland]. One school abroad had a dean who advised students hoping to return to Canada and helped students navigate electives: “*[Medical School] has a fantastic vice dean…So she helps out a lot in regards to guidance for how to apply, where to apply, where my chances would be higher, what they’re looking for on my personal statements and stuff like that, where I should be doing my electives*” [1; Residency; Ireland].

Many participants had personal connections to physicians who assisted them by helping them access electives in Canada (“*I had a family friend who is a family doctor and I did an unofficial elective with him, but if you don’t have connections, it’s quite difficult, and to get into the specialty that you want to go to, it’s like, a huge advantage*” [6; Pre-residency; Ireland]), liaise with regulatory officials (“*my father is a physician … He knew someone on the [licensing board] and just sort of explained my situation and that helped to speed up that whole verification of my residency certificate”* [14; Post-residency; Grenada]), or navigating the post-graduate medical education system (“*I suppose my connections and my networks in the medical community here will be of assistance whenever it’s time for me to write residency applications*” [27; Pre-residency; Ireland]. These participants felt that their connections to the medical system gave them an advantage over other internationally-trained students.

## Discussion

Canadians choose to study medicine abroad to avoid the potential and actual road-blocks to studying medicine in Canada (including pre-requisite undergraduate degrees, and non-competitive or unsuccessful applications). Key factors (residency matching rates, location, costs) that influence where CSA choose to study reflect the messages on offshore medical school publicity materials that appeal to students’ sense of injustice at not being admitted to medical school in Canada [2, 3]. CSA reported that they did not fully appreciate the challenge of being an IMG and securing a post-graduate medical residency position in Canada, particularly in terms of additional costs, practical and logistical challenges, and the toll on their mental health. CSA have many informal and formal supports, in both Canada and in their medical school host countries, to help them navigate the process of obtaining a medical residency position in Canada.

The number of CSA and the pathways by which they return to Canada remain unclear. CSA who are unable to obtain a residency position in Canada may return to Canada after completing post-graduate training elsewhere but will still have to meet the credentialing and licensing requirements for IMG. Alternatively, they may need to emigrate to work as a physician. Analyses of administrative medical education data report that less than one-third of CSA obtain a post-graduate training position in Canada [24, 25] and that roughly one-third of CSA who apply for a residency position in North America are unable to obtain a position in either Canada or the US [24]. A recent study of Canadian medical students who did not match to a post-graduate position noted the dire financial (termination of line-of-credit), licensing (inability to continue clinical training), and mental health consequences [26]. It is likely that CSA who are unable to obtain a residency position face similar hardships [27].

Our study highlights the perceived differences in the format of medical education in international schools. CSA have less opportunity for hands-on clinical rotations at these schools than in Canada. Medical schools in the Caribbean have contracted hospital systems in the US to provide their students with clinical clerkship rotations, driven in part by the criticisms of the lower performance of offshore graduates in national credentialling examinations and post-graduate training match rates [2, 12–14]. In Canada, CSA are able to complete clinical clerkship rotations during medical school, which gives CSA an advantage over immigrant IMG in the residency matching process in Canada [7, 11].

Study findings also highlight the relative privilege of CSA in comparison to other immigrant IMG who compete for post-graduate training positions in Canada. In addition to being able to afford the expense of an international education, some study participants reported having connections to physicians that provided them with social capital and ‘insider knowledge’; findings that are consistent with a previous survey of CSA that found that one in five CSA had a physician parent [18] and interviews with medical educators [11].

### Limitations

Interview data may be subject to recall, social desirability, and selection bias [28, 29]. We were unable to interview CSA who had not been able to secure a residency position, who had failed medical school, or who had emigrated. Thus, the data likely presents an optimistic portrayal of CSA experiences. We interviewed few students from Australia, the Caribbean, or Poland (where many CSA attend medical school) despite concerted recruitment efforts. CSAs’ experiences may vary by region, especially in terms of matching to residency positions in Canada or the US.

### Future research

In 2019, the examinations required for IMG post-graduate applicants changed (eliminating the computer-based Medical Council of Canada Evaluating Exam and requiring the in-person NAC OSCE) [30, 31]. The COVID-19 pandemic created travel disruptions that made travel to Canada (for medical electives, interviews and/or examinations) more difficult. Future research should examine the impacts of these policy changes and the pandemic on the characteristics of IMG (CSA and immigrant IMG) who applied and were admitted to post-graduate programs in Canada. Additionally, future research should examine alternate pathways that IMG are able to qualify to work in Canada, such as through fellowship training or recruitment at later career stage (after completing residency training and/or working elsewhere).

## Conclusions

The study provides information for Canadians considering studying abroad as well as medical school educators selecting applicants for post-graduate programs, and health workforce planners. Our study is among the first qualitative studies to examine CSA experiences. Canadian students may not be aware of the many potential risks of training abroad and the challenging steps required to return to Canada to practice medicine. Medical educators should anticipate training needs of graduates from offshore medical programs that provide less hands-on clinical experience than Canadian medical schools. Health workforce planners in Canada and elsewhere should also consider the implications of this large pool of trainees seeking to join the physician workforce. Offshore medical schools remain a popular choice for Canadian trainees, have become a steady source of new physicians, and have implications for post-graduate medical education and health workforce planning in Canada and elsewhere.

## Electronic supplementary material

Below is the link to the electronic supplementary material.

**Supplementary Material 1:** Appendix A: Interview Questions for CSA Participants

## Data Availability

The datasets analysed during this study are not publicly available due to the need to maintain participant confidentiality; however, a portion of these data may be available from the corresponding author on reasonable request.

## List of Abbreviations

CaRMS: Canadian Resident Matching Service
CSA: Canadians who study medicine abroad
IMG: International medical graduate
MCAT: Medical College Admission Test
NAC OSCE: National Assessment Collaboration Objective Structured Clinical Examination
UK: United Kingdom
US: United States
USMLE: United States Medical Licensing Exam
