# Supplementary material for: “I wish I had known what I was getting into”: a qualitative study exploring the experiences of Canadians who study medicine abroad
Source: BMC Med Educ. 2023 May 24;23:376. doi: 10.1186/s12909-023-04367-1 (PMC10206541; doi:10.1186/s12909-023-04367-1)
Supplement: Supplementary file 1 — Supplementary Material 1: Appendix A: Interview Questions for CSA Participants [file 12909_2023_4367_MOESM1_ESM.docx]

**Appendix A: Interview Questions for CSA Participants**

1. To begin, I have a couple of questions about your background?
   1. Where were you born?
   2. Where did you grow up?
   3. Where did you attend school/complete your undergraduate training?
   4. Are you a Canadian citizen? When did you become a citizen?
   5. Where did you attend medical school?
      1. Probe: name of the institution
      2. Probe: country/city of the institution in?
2. What made you decide to go to medical school in ___________ (country/name)
   - 1. Probe: admission requirements,
     2. Probe: difficulties with acceptance into Canadian schools,
     3. Probe: financial considerations
     4. Probe: geographical preferences
3. How do you feel about your experience of in medical school in _________ (country)
   - 1. Probe: positive
     2. Probe negative
4. At what stage are you in getting your license to practice in Canada
   - 1. Probe: which exams taken/passed
     2. Probe: residency – applied, accepted, in process, completed
     3. Probe: currently practicing - full licence, provisional/restricted license
5. What difficulties, if any, did you experience in getting to this point?
   - 1. Probe: passing exams
     2. Probe: securing residency training
6. Was there anything that made getting to this point in the licensing process easier? How did it help you?
   - 1. Probe: clinical rotation in Canada during med school
     2. Probe: skills assessment/enhancement courses
     3. Probe: examination preparation
     4. Probe: accepting residency positions in less appealing fields
     5. Probe: personal network
        1. With whom?
        2. What did they do?
7. What resources should be available to help CSA through the licensing process?
   - 1. Probe: clinical rotations in Canada during med school
     2. Probe: skills assessment/enhancement courses
     3. Probe: examination preparation
     4. Probe: dedicated residency positions in less appealing fields
     5. Probe: personal network
   1. Should these be different than those available for other IMG? Why?
8. Should the requirements or process for CSA be different than they currently are? And if so how?
   - 1. Probe: Success rates on examinations,
     2. Probe: language proficiency,
     3. Probe: cultural knowledge,
     4. Probe: likelihood of retention in rural areas
     5. Probe: Canadian resident/citizen
   1. Should they be different than those for other IMGs? Why?
9. Is there anything else you would like to add?
